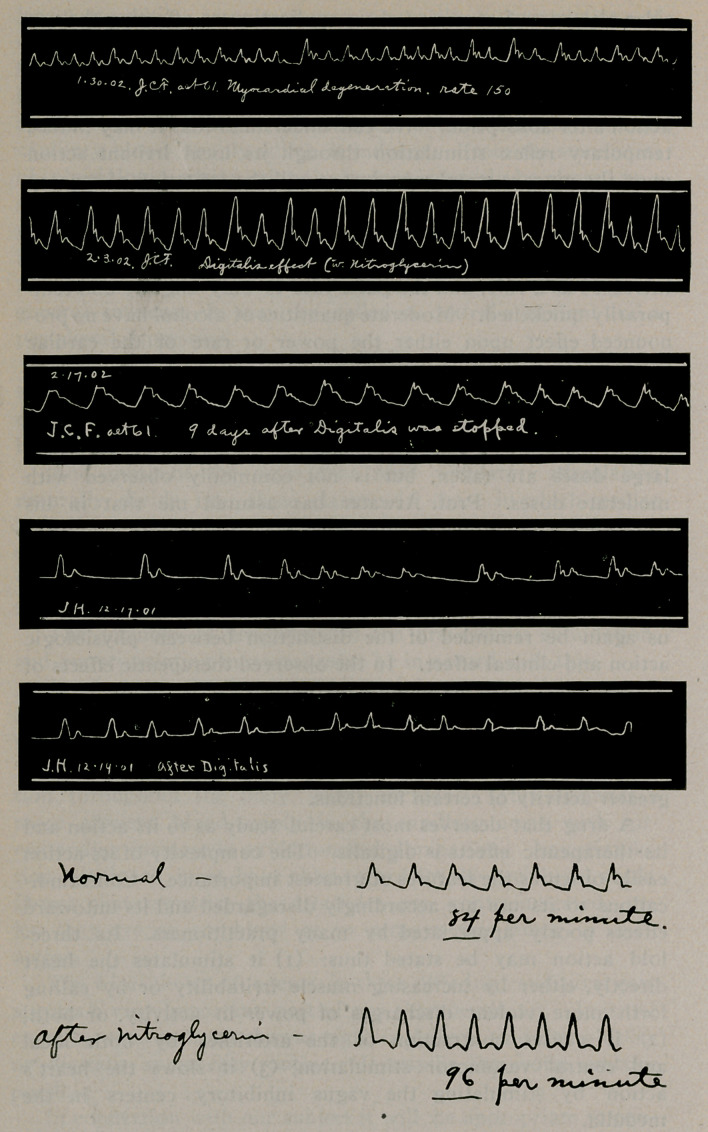# Stimulation1Read before the section on medicine, Buffalo Academy of Medicine, February 10, 1903.

**Published:** 1903-06

**Authors:** Eli H. Long

**Affiliations:** Buffalo, N. Y., Professor of Therapeutics, Medical Department, University of Buffalo. 1335 Main Street


### Stimulation.[1]

By ELI H. LONG, M.D., Buffalo, N.Y.,
Professor of Therapeutics, Medical Department, University of Buffalo.

THE perennial interest in whatever pertains to emergency measures justifies an occasional reconsideration of our ideas of so important a subject as stimulation. In attempting a definition of the term we meet with difficulties that have increased with more exact knowledge of physiology, for it has been found that certain changes produced by agents credited with stimulant properties are primarily and essentially depressant; and, on the contrary, certain sedative effects upon organs may be the physiologic effect of a true stimulation. It is difficult to restrict the term to the simple idea of excitation of an organ to increased functional activity; *i. e.*, stimulation in a physiologic sense. And, on the other hand, it seems improper to permit the secondary effects alone to determine what shall be regarded as stimulation. It is largely a question of reconciling physiologic action of drugs with their apparent clinical effects.

In order to illustrate the difficulties, let us take two well-

1. Read before the section on medicine, Buffalo Academy of Medicine, February 10, 1903.

known drugs, aconite and alcohol. Aconite is regarded as a typical heart depressant, as judged by its effects, as it at once slows and weakens the heart's action. But it is found that these effects are secondary, being brought about by the stimulation of the inhibitory vagus center in the medulla, which results in a restraint of the action of the heart and a relaxation of its muscular tone. It might be entitled to be called physiologically a stimulant, but clinically it must be regarded as a sedative. On the other hand, alcohol, judged simply by its apparent effects, has held a prominent place as a stimulant, but experimental evidence goes to show that certain of the apparent stimulant effects, such as hilarity and garrulity, are really due to a removal of the restraint of the controlling cerebral functions by the depressant action of the drug.

There is likely to be for some time to come conflicting opinions held by physiologists and pharmacologists on the one hand, and clinicians on the other, concerning the true place of this and some other substances, but a final agreement must be possible if we approach the matter as we ought any scientific question that requires observation, experiment and unprejudiced deductions from observed facts, for its solution.

In order to clear away inherited or preconceived views for the time being, let us question whether we should use the term stimulation as meaning always a single definite and invariable kind of influence or phenomenon. Let us be prepared to recognise a restricted physiologic meaning of the term and possibly also a broad clinical meaning, based upon the complex results of the action of remedies as observed in disease.

In the study of the practical values and uses of remedies we employ several related terms which are too often confused. Physiologic action, physiologic effect and therapeutic effect are terms distinct in meaning, and they should be properly understood. The action and the effect of a drug cannot be the same. The action may be obscure; the effect must be apparent. The action of a drug consists of a modification of conditions, chemic, thermic, electric, or structural, which determines an alteration of function. This alteration, when apparent, is known as the effect of the drug. Within the limit of normal function this effect is physiologic, while an alteration beyond the normal is a toxic effect. Therapeutic effect means an alteration that relieves a symptom or condition of disease, and presupposes a pathologic state.

In the analysis of the physiologic action of stimulants in the laboratory, we are dealing chiefly with an increased irritability of cells, or with a more ready discharge of their peculiar power. There is this objection to calling either of these factors stimulation—the physiologist is not working in the proper field of stimulation. He is using tissues in as nearly normal condition as possible. In order to develop this thought we must appreciate the limitations of stimulation.

The indication for employing stimulants is, in general, any depression of a function to a degree that may be regarded as below the physiologic minimum of its activity. We recognise that every organ has a certain range of action that may be called physiologic, within which it reacts to the work demanded of it, by increasing or lessening its activity. Functional activity, therefore, is a variable quantity, influenced on the one hand by the strength and nutritive resources of an organ, which are opposed on the other hand by the amount of work imposed upon it. The physiologic minimum of activity, therefore, must vary as modified by these influences; but it may be defined to be the minimum of efficiency of a function under existing conditions. Now as long as an organ is working efficiently within its physiologic range it needs no stimulation. But when, either from its own inability or from excessive demands made upon it, its activity falls below its physiologic minimum, then stimulation may be employed to compel an extra expenditure of energy in enforced activity.

It is, moreover, observed that normally-acting organs do not show much response to stimulants, but that those whose action is deficient respond well. When an organ is doing all the work that is required of it, it is difficult to force its action; but when a need of increased work is present and a stimulant is applied, there appears to be a coöperation of influences, the increased irritability or the more powerful impression contributed by the applied stimulant enabling the organ to respond to the need of increased work, which, after all, is its normal stimulus.

The physiologist, therefore, is studying biologic reactions which are made to serve the need of stimulation only in abnormal conditions. The classic definition of a stimulant is—an agent that increases the activity of a function. If we turn to the clinical side of the question we must at least qualify this statement by stating that we seek not always rapidity, but always efficiency of a function. With this as a goal, it must appear that we are seeking to secure effects rather than precise medicinal action. In one case efficiency of a function will be secured by temporary rest; in another by restraint; in another by removal of restraint; in another by increasing reserve power; in another by positive stimulation of an organ. Herein lies the difficulty of a scientific use of the term in its broad sense. But inasmuch as we are not yet prepared to discard it, let us attempt to understand how our desired therapeutic effects are brought about.

In the regular exercise of most functions in the living body four factors are potent: (1) reserve energy; (2) irritability); (3) activity or discharge of power; (4) restraint or inhibition.

Assuming that reserve energy is sufficient, an increase of the efficiency of a function may be secured by modifying any one of the three other factors. There may be a diminution of reserve power, by reason of hemorrhage, damage to blood and tissues by poisons either from without or from within the system, or from various other causes. Here, whatever contributes to a restoration of the nutritive qualities of the blood and the volume of fluid in circulation will serve our purpose. It is often observed after hemorrhage that the restoration of volume by hypodermic or intravenous use of normal saline solution will produce a much better action of the heart. Several factors may be concerned in producing this result. Better filled vessels mean a better nourished heart; better distention of the ventricles with fluid calls forth a better contraction; while the saline itself may increase the irritability of the heart muscle. The effect is not purely a stimulation, for we are contributing not only to functional activity but to reserve power as well.

The following illustrations of drug action as related to improvement of the circulation are given:

First, strychnin acts chiefly by increasing the irritability of nerve tissues. It does not cause the discharge of power but makes the reflex center more responsive to normal stimuli. It might therefore be called a potential stimulant. Even in poisoning by strychnin the convulsions do not originate independently of external stimuli, as is indicated by the fact that they do not occur in a strychninised frog, when the surface of the body has been previously cocainised in order to prevent the stimuli being carried inward from the surface. Herein lies the superiority of strychnin as a general stimulant—that it does not cause an exhausting discharge of power. It simply places the nervous system and possibly the muscles in a more responsive condition to the needs of functional activity. Its application is apparent in such diseases as pneumonia, diphtheria and typhoid fever, where stimulation is desired without exhausting either reserve energy or irritability. Digitalis, in moderate doses, probably also acts by increasing the irritability of the heart muscle, without causing much extra discharge of energy.

Second, another kind of stimulation is that which increases activity by causing either more frequent or more violent discharges of energy in the line of the particular function affected. Thus, the heart will respond to any considerable irritation of sensory nerves by whatever agent produced. Pain will often induce a rise of arterial pressure. Irritation by the faradic current will give the same result. Stimulation of this nature is brought about in an indirect or reflex manner. The so-called diffusible stimulants, such as alcohol and ammonia, probably induce much of their effect in this way, being diffusible in effect, rather than in action. In the relation of their physiologic action to reserve energy they may be regarded as kinetic stimulants, in that they cause a more rapid change of reserve into kinetic energy. Caffeine, and drugs of the digitalis group, in full doses, belong to this class, caffeine causing a more rapid heart action and digitalis more forcible contractions. By causing an increased discharge of energy these agents tend toward exhaustion and their abuse must always be guarded against. They have in their excessive action the possibility of defeating the very end sought to be gained by stimulation. Before employing kinetic stimulants, the question should always be raised whether their temporary value will compensate for the extra demand they make upon the reserve energy; and it is plain that their continuous use must be carefully supervised.

Third, the action of those agents that produce a stimulant effect of a certain kind by paralyzing or depressing inhibition, is very interesting. Atropin and glonoin are the typical drugs of this class. Their essential action is a depression of the vagus influence upon the heart, whereby the accelerator influence becomes relatively prominent, with an increase of the pulse rate. They differ in action in two respects; glonoin paralyzes the vagus center in the medulla, while atropin paralyzes its cardiac terminals, the effect upon the heart being the same; and, peripherally, their circulatory effects are opposite, glonoin inducing dilatation of arterioles by a direct paralyzant action upon their muscular coat, while atropin causes primarily a constriction of arterioles by central vaso-motor stimulation. Any direct stimulation of the heart by either is uncertain. As circulatory stimulants, the value of atropin must depend upon its power to slightly raise arterial pressure and that of glonoin upon the freer capillary blood supply which it induces. From the standpoint of physiologic action glonoin cannot properly be classed as a stimulant, as its action is essentially paralyzant, although we rank its effects as most valuable in increasing the peripheral blood supply in suitable cases. Atropin has a definite stimulant action upon centers in brain and medulla, therefore in its general influence it may be called a central stimulant and a peripheral depressant.

From the viewpoint of clinical results we usually seek by stimulation to secure one or more of the following: (1) more efficient heart action; (2) increase of arterial pressure; (3) better blood supply to the capillary areas.

And most of the cases that need circulatory stimulation naturally divide into three classes: (*a*) those in which cardiac irritability of activity is interfered with by intoxications of various kinds; (*b*) those in which serious valvular disease prevents compensation without aid; (*c*) those in which arterial sclerosis is antecedent to myocardial failure.

The keynote of treatment in the first class is elimination, and our stimulating measures must be in accord with this.

In the second class the chief needs are temporary rest to the heart, with maintenance of its nutrition. Our stimulation must respect these needs.

In the third class the great need is to promote cellular nutrition throughout the body, heart included. This demands a more efficient blood-supply in the capillary areas, with corresponding increase in oxidation and elimination. Any stimulation employed must have chief regard for the peripheral needs. Drugs that constrict the arterioles are contraindicated, unless they are so combined as to remove this factor in their action.

The wise choice of agents in these differing conditions constitutes a very important part of our therapeutic art. It is not enough, when a heart showns signs of failure, to order whiskey or digitalis as an easy routine. It may be the worst practice possible.

In connection with our subject it will be appropriate to consider also some features of drug application as affecting the use of several of the more commonly used stimulants.

To consider, briefly, the place of alcohol as a stimulant, we must admit that we face the difficulty of uncertainty as to its action after absorption. We can understand that it may induce temporary reflex stimulation through its local irritant action upon the mucous membrane, just as will the irritation of sensory organs by faradism. However, observations as to such action in dogs do not show any very decided results, as the blood pressure tracings will indicate. Arterial blood pressure is not increased as a rule, and the pulse rate is only slightly and temporarily quickened. Moderate quantities of alcohol have no pronounced effect upon either the power or rate of the cardiac systols, while full doses weaken the auricular contraction decidedly and the ventricular contraction in lesser degree. (Cushing). Its action upon the arterioles is indefinite. It is usually classed as a vasodilator and this effect is apparent when large doses are taken, but is not commonly observed with moderate doses. Prof. Atwater has assured me that in his calorimetric experiments with men taking moderate quantities of alcohol, he has not observed flushing of the skin. Notwithstanding these observations some clinicians assert its stimulant effects upon the circulation. In the face of these differences let us again be reminded of the distinction between physiologic action and clinical effect. In the observed therapeutic effects of alcohol several factors may be involved. Since the influence of inhibition as a factor in producing a depressed state of the system is admitted, it may be argued that the useful effects of alcohol are partly due to its power to weaken inhibition, thus permitting greater activity of certain functions.

A drug that deserves most careful study as to its action and its therapeutic effects is digitalis. The complexity of its action easily obscures the features of greatest importance. Contraindications to its use are accordingly disregarded and its untoward effects poorly appreciated by many practitioners. Its threefold action may be stated thus: (1) it stimulates the heart directly, either by increasing muscle irritability or by calling forth more violent discharges of power in activity, or both; (2) it causes contraction of the arterioles by both local and central vasomotor stimulation; (3) it slows the heart's action by stimulating the vagus inhibitory centers in the medulla.

1.30.02. J.C.F. aet 61. Myocardial degeneration. rate 150

2.3.02. J.C.F. Digitalis effect (w. Nitroglycerin)

2.17.02

J.C.F. aet 61. 9 days after Digitalis was stopped.

J.H. 12.17.01

J.H. 12.14.01 after Digitalis

Normal –
84 per minute.
after nitroglycerin –
96 per minute

Whenever digitalis is indicated its direct stimulant action is always desired. Its inhibitory stimulant action may be desired, while its vasoconstrictor action is often positively harmful. Its stimulant action upon the kidneys is uncertain, although it is found that with dogs a pure digitalin, in large dose, positively lessens the secretion of urine. It is generally accepted that the whole drug has a diurectic effect in certain cases through the rise of arterial pressure which it induces.

The several modes of action of digitalis are too often regarded as of equal prominence and importance. We ought to rate its first effect, that of direct heart stimulation, as its best effect; and we may usually have this action of the drug from moderate doses, while any marked slowing of the heart is a later effect, often scarcely seen except with large doses. One tracing here shown gives an actual increase in the pulse rate under digitalis. There may be varying indications for securing an altered rate of the pulse, as in some cases of aortic incompetency and mitral stenosis; but, according to Cushing, the inhibitory stimulation not only slows but relaxes the muscular fibers, and weakens systole. This statement is supported by bringing into comparison the effects of aconite, which has precisely the same inhibitory stimulant action as digitalis without any direct effect upon the heart, the result being slowing and relaxation of the organ.

The vasoconstrictor action is the part of the drug's influence that is most frequently undesirable. A close study of the relation of arterial sclerosis primarily to nutrition in the capillary areas and secondarily to nutrition of the heart, will convince one of the supreme importance of good reactive power in the muscular coat of the smaller blood vessels. By reactive power we mean the ability to either dilate or contract easily as the needs of the capillary system requires. Theoretically, we suppose that in arterial sclerosis this power of reaction is much reduced, and that cellular nutrition suffers accordingly. In the use of digitalis in the third class of cases previously mentioned, the vasoconstrictor action may, therefore, interfere with the already diminished power of reaction in the arterioles to such a degree that autointoxication may be favored and our purpose in medication defeated. In many of these cases it is imperative to remove the vasoconstrictor action of digitalis when this drug is employed. This can be done by combining it with one of the nitrites, either glonoin or sodium nitrate.

Another frequent advantage of such a combination is the removal or diminution of the inhibitory influence which may be relatively too prominent, because of the diminished irritability of the heart muscle; for either faulty nutrition or myocardial degeneration may reduce the muscle irritability and give us the slow heart, so frequently met with in advanced life which is doubtless a frequent cause of sudden death. The fact of loss of muscular irritability in old hearts will bear emphasis and will often modify our treatment.

1335 Main Street.

## Figures and Tables

**Figure f1:**